# Adherence to Internet-Based and Face-to-Face Cognitive Behavioural Therapy for Depression: A Meta-Analysis

**DOI:** 10.1371/journal.pone.0100674

**Published:** 2014-07-16

**Authors:** Wouter van Ballegooijen, Pim Cuijpers, Annemieke van Straten, Eirini Karyotaki, Gerhard Andersson, Jan H. Smit, Heleen Riper

**Affiliations:** 1 Department of Clinical Psychology, VU University Amsterdam, Amsterdam, The Netherlands; 2 Department of Psychiatry, VU University Medical Centre/GGZ inGeest, Amsterdam, The Netherlands; 3 EMGO Institute for Health and Care Research, VU University Medical Centre, Amsterdam, The Netherlands; 4 Department of Behavioural Sciences and Learning, Swedish Institute for Disability Research, Linköping University, Linköping, Sweden; 5 Department of Clinical Neuroscience, Karolinska Institute, Stockholm, Sweden; 6 Leuphana University, Lüneburg, Germany; University of Granada, Spain

## Abstract

**Background:**

Internet-based cognitive behavioural therapy (iCBT) is an effective and acceptable treatment for depression, especially when it includes guidance, but its treatment adherence has not yet been systematically studied. We conducted a meta-analysis, comparing the adherence to guided iCBT with the adherence to individual face-to-face CBT.

**Methods:**

Studies were selected from a database of trials that investigate treatment for adult depression (see www.evidencebasedpsychotherapies.org), updated to January 2013. We identified 24 studies describing 26 treatment conditions (14 face-to-face CBT, 12 guided iCBT), by means of these inclusion criteria: targeting depressed adults, no comorbid somatic disorder or substance abuse, community recruitment, published in the year 2000 or later. The main outcome measure was the percentage of completed sessions. We also coded the percentage of treatment completers (separately coding for 100% or at least 80% of treatment completed).

**Results:**

We did not find studies that compared guided iCBT and face-to-face CBT in a single trial that met our inclusion criteria. Face-to-face CBT treatments ranged from 12 to 28 sessions, guided iCBT interventions consisted of 5 to 9 sessions. Participants in face-to-face CBT completed on average 83.9% of their treatment, which did not differ significantly from participants in guided iCBT (80.8%, *P*  =  .59). The percentage of completers (total intervention) was significantly higher in face-to-face CBT (84.7%) than in guided iCBT (65.1%, *P* < .001), as was the percentage of completers of 80% or more of the intervention (face-to-face CBT: 85.2%, guided iCBT: 67.5%, *P*  =  .003). Non-completers of face-to-face CBT completed on average 24.5% of their treatment, while non-completers of guided iCBT completed on average 42.1% of their treatment.

**Conclusion:**

We did not find studies that compared guided iCBT and face-to-face CBT in a single trial. Adherence to guided iCBT appears to be adequate and could be equal to adherence to face-to-face CBT.

## Introduction

A growing number of self-help interventions for depression are available on the internet. Internet-delivered self-help programmes can be an effective treatment for depression [1], [2]. These interventions can be roughly divided into guided and unguided interventions. Unguided interventions are fully automated self-help programmes without any therapist support, e.g. [3]–[5]. Guidance in guided self-help interventions is usually conducted via email by a therapist or coach, e.g. [6]–[8]. In contrast to a therapist, a coach does not need to be a licensed professional, but can also be a trained volunteer or a professional with lower levels of education. Guided internet interventions are generally considered to be more effective in reducing depressive symptoms than unguided ones [2], [9], [10].

Most internet interventions for depression consist of internet-delivered cognitive behavioural therapy (iCBT), or related therapies, such as problem solving therapy [8], [11], even though internet interventions might also be based on other therapeutic orientations, e.g. psychodynamic therapy [12]. Guided iCBT has proved to be an effective form of treatment for depression [1], [2] and acceptable for both patients and professionals [13]–[15]. However, internet interventions can be associated with substantial attrition [16], [17]. The attrition or adherence rates for guided iCBT for depression have not yet been systematically studied, while they are an important measure of acceptability and could be related to treatment outcome [18].

Attrition can be defined in a number of ways. Previous studies have discussed terms like therapy drop-out and premature discontinuation [19], premature termination [20], non-usage attrition [21], (non-)persistence [22], and (non-)adherence [16]. Although all these terms describe the extent to which an individual completes the treatment, the definitions differ. For the present study, we will use the terms adherence and non-adherence to describe the extent to which individuals are exposed to the content of the intervention [16]. This can be operationalised by dividing the mean amount of completed sessions or modules by the maximum amount of sessions or modules. This definition can apply both to face-to-face treatment and internet-based treatment. We will add to this definition that participants allocated to an intervention who do not start the treatment should also be included in the non-adherence rate. There are two reasons for this addition. First, in randomised trials, it is important that all randomised participants are included in intention-to-treat analyses to preserve the unbiased comparison between groups allowed by randomisation [23]. Second, individuals who proceeded as far as allocation to a treatment can be regarded as having the intention to be treated.

The first meta-analysis of non-adherence to psychotherapy in its traditional face-to-face setting showed that about 50% of those that started their treatment completed it [24]. However, in a new meta-analysis among more recently published studies (1990-2010), the percentage of completers was 80.3% [19]. Cognitive behavioural therapy (CBT) performed slightly better than the general average (81.6%), as did therapies for patients with mood disorders (82.6%). It is important to note that both meta-analyses [19], [24] included only participants who had started the intervention. Patients who did not show up for their first session were excluded from the analyses. Although the proportion of completers according to our definition would, therefore, be lower, it is evident that adherence to CBT for depression could be relatively high, which is further underlined by other studies [25].

To date, the adherence to guided iCBT for depression has not been systematically studied. With regard to computerised CBT (a broader definition than iCBT, for instance including a self-help course on CD-ROM) for depression, the drop-out rate averages 31.75% [14]. However, the definition of drop-out was quite diverse among the sixteen studies in the review by Kaltenthaler [14] and comprised both non-adherence to treatment and non-response to follow-up measures. Waller and Gilbody [26] found that a median of 56% treatment starters completed a full course of computerised CBT. In their meta-analytic review, Richards and Richardson [2] found that adherence to computerised CBT was associated with type of guidance. Overall, the percentage of completers was 43%. For therapist-guided computerised CBT interventions, it was 72%, for administrative support 65.2% and for no support 26%. These rates of completion in computerised CBT cannot be easily generalised to guided iCBT. Computerised CBT may also include therapy sessions on CD-ROM or DVD, or on stand-alone computers in a clinical practice, and guidance may be given by face-to-face contact, telephone or email. iCBT programmes, on the other hand, are followed completely on the internet, and can be guided by email, chat or telephone. Additionally, meta-analyses and reviews to date have focused mainly on drop-out rates instead of the extent to which individuals are exposed to the content of the intervention. That is, it was not taken into account whether non-completers dropped out early on or nearly completed the intervention.

In sum, guided iCBT is a promising treatment for depression, but to date, the overall adherence to this type of treatment is unknown. It is also unknown how the adherence to guided iCBT compares with face-to-face CBT for depression. The present study is a meta-analysis investigating the adherence to guided iCBT self-help interventions, and comparing these with adherence to individual face-to-face CBT interventions.

## Method

### Identification and selection of studies

We used an existing database that is aimed to include all randomised trials of the psychological treatments of depression. This database has been described in detail elsewhere [27] and has been used in a series of meta-analyses (www.evidencebasedpsychotherapies.org). The extracted data are available on request. The database contains studies from 1974 and has been continuously updated through comprehensive literature searches up to January 2013. In these searches, 14,164 abstracts were examined in PubMed (3638 abstracts), PsycInfo (2824), Embase (4682) and the Cochrane Central Register of Controlled Trials (3020). The abstracts were identified by combining terms indicative of psychological treatment and depression, using both MeSH-terms and text words. Details of the search strings are presented in a previous study [27]. Some grey literature was included as well, such as PhD theses that contained material that had not been published elsewhere. Due to time constraints, further unpublished material was not searched for, and authors of studies describing incomplete data were not contacted. Non-randomised studies are also relevant when examining adherence, but were excluded. These are few in number and cannot be compared validly with randomised studies, because randomised allocation may have an effect on adherence. For the current study, previous reviews and meta-analyses of computerised CBT and face-to-face CBT for depression were also checked for additional studies.

For the present meta-analysis, we included studies on CBT among depressed adults (18+ years), including student samples and elderly samples. 'Depressed' was defined as major or minor depressive disorder according to a diagnostic interview or an elevated level of depressive symptoms. Depressive symptoms could be indicated by a score above a cut-off point on a validated self-report depression scale like the Beck Depression Inventory. We only included trials in which at least one treatment group was offered guided iCBT or individual face-to-face CBT. CBT was defined as treatment in which cognitive restructuring is the core element, commonly based on the manual developed by Beck, Rush, Shaw & Emery [28], or treatment in which cognitive restructuring is an important component, but where components such as behavioural activation, social skills training, relaxation, or coping skills also have a prominent place. An example of the latter approach is the Coping with Depression course [29]. Guided iCBT was defined as internet-based self-help CBT that includes coaching or guidance by email, chat or telephone. Face-to-face CBT was defined as CBT delivered face-to-face by a therapist to an individual. When a study contained several treatment groups who were offered CBT or iCBT, these were coded as separate, independent groups. The selection of the studies was conducted by the first author.

We excluded studies that examined CBT for patients with comorbid somatic conditions (e.g. diabetes) or addictions, and studies on relapse prevention. Also excluded were studies on CBT delivered by book (bibliotherapy), CD-ROM, email or telephone, unguided iCBT, studies in which the CBT intervention was combined with pharmacotherapy, and studies on group CBT. Group CBT was an exclusion criterion, because guided iCBT is individual and, therefore, more comparable with individual face-to-face therapy. To increase the comparability between studies on guided iCBT and studies on individual face-to-face CBT further, we also excluded studies based on publication year and recruitment method. Research on iCBT emerged in the early 2000s, so we excluded all studies that were published before the year 2000. Because participants in iCBT are often self-referred [26], we excluded studies on inpatients and only included studies that recruited their sample largely or entirely from the community, i.e. by means of advertisements in newspapers or magazines, banners on websites, or large scale mailings.

No language restrictions were applied.

### Coding and data extraction

The main outcome variable of our analyses was the average number of modules, lessons or treatment sessions (and standard deviation) that were completed by the participants, divided by the total number of sessions to obtain a proportion or percentage. In some cases, these values were not reported, but could be calculated based on the flow chart, or could be estimated. Estimates were conservative, expecting low adherence, to avoid overestimating the overall adherence rates. For example, a study could describe an intervention of eight sessions with one session every week, and report that two participants dropped out during the first four weeks. In that case, we considered these two participants to have completed one session. When possible, we created a data file containing the number of completed sessions for each participant in a particular study based on all information extracted from that particular paper. Means and standard deviations were then calculated using SPSS 19. The secondary outcome variables concerned the percentage of intervention completers, of which we coded both the 100% and 80% or more intervention completion rate. Studies on adherence usually focus on the amount of intervention completers, so we included these outcome measures for comparability with other studies. The 100% completion rate was defined as the number of participants who had completed all sessions or modules divided by the number of participants allocated to the intervention. We also coded the number of participants that had completed at least 80% of the intervention, i.e. 80% of the total number of sessions. These participants may not have completed the entire intervention, but were exposed to a substantial part of the treatment content. When the 80% treatment completion was not reported and could not be inferred, we analysed the number of participants who had completed 100% of the intervention for that study, because those are the participants of whom we can be sure to have completed at least 80%.

We included in our analyses all participants who had completed the pre-treatment measures and were allocated to a CBT condition, regardless of whether or not the participant started the intervention. In half of the included studies on face-to-face CBT, participants who had completed a substantial part of the intervention were defined as completers, for example participants who had completed at least twelve sessions of a twenty-session treatment protocol. In some of these cases, we were still able to estimate the adherence rates by using the respective authors' definition of completers. For these studies we still used the maximum amount of sessions, which is usually the number specified in therapy manuals, to calculate the proportion of sessions completed. When no other information was available, completers as defined by a study's authors were assumed to have completed the entire therapy protocol, i.e. for our analyses they were assumed to have completed the maximum amount of sessions. This assumption could overestimate adherence to face-to-face CBT.

Data abstraction from the studies was conducted by two independent raters (WvB and EK). Differences were discussed until consensus was reached.

### Quality assessment

The validity of included studies in meta-analyses is usually assessed, for example, by using criteria of the ‘Risk of bias’ assessment tool, developed by the Cochrane Collaboration [30]. This tool assesses possible sources of bias in randomised trials. In the current study, criteria pertaining to randomisation, allocation concealment and blinding of participants, personnel and outcome assessors were not assessed. These criteria were not relevant to our study, because we did not assess effectiveness or outcomes of the interventions. We assessed studies for the quality criterion pertaining to adequate dealing with incomplete outcome data. This involved examining whether all participants allocated to the intervention were included in the analyses and how missing data were analysed. We also coded whether rates of intervention adherence and study drop-out were reported. The quality assessment was conducted by two independent reviewers (WB and PC), and disagreements were resolved by discussion.

### Meta-analyses

The heterogeneity between studies was calculated using the *I^2^*-statistic. This *I^2^* is expressed in percentages. A value of 0% indicates no heterogeneity, 25% indicates low, 50% indicates moderate, and 75% indicates high heterogeneity [31]. We also report the significance of the Q-statistic, which indicates whether the heterogeneity was significant or not. Since we expected considerable heterogeneity, we decided to perform our analyses using a random effects model. In this model, the included studies differ not only because of the random error within studies (as in the fixed effects model), but also because of true variation from one study to the next.

Each outcome measure was pooled in the subgroups face-to-face CBT and guided iCBT. We performed subgroup analyses to compare adherence rates between these groups. These analyses were conducted according to the mixed effect model. In this model, studies within subgroups are pooled with the random effects model, while tests for significant differences between subgroups are conducted with the fixed effects model. All analyses were performed with Comprehensive Meta-Analysis (CMA; version 2.2.021).

## Results

### Selection and inclusion of studies

**Figure 1:**
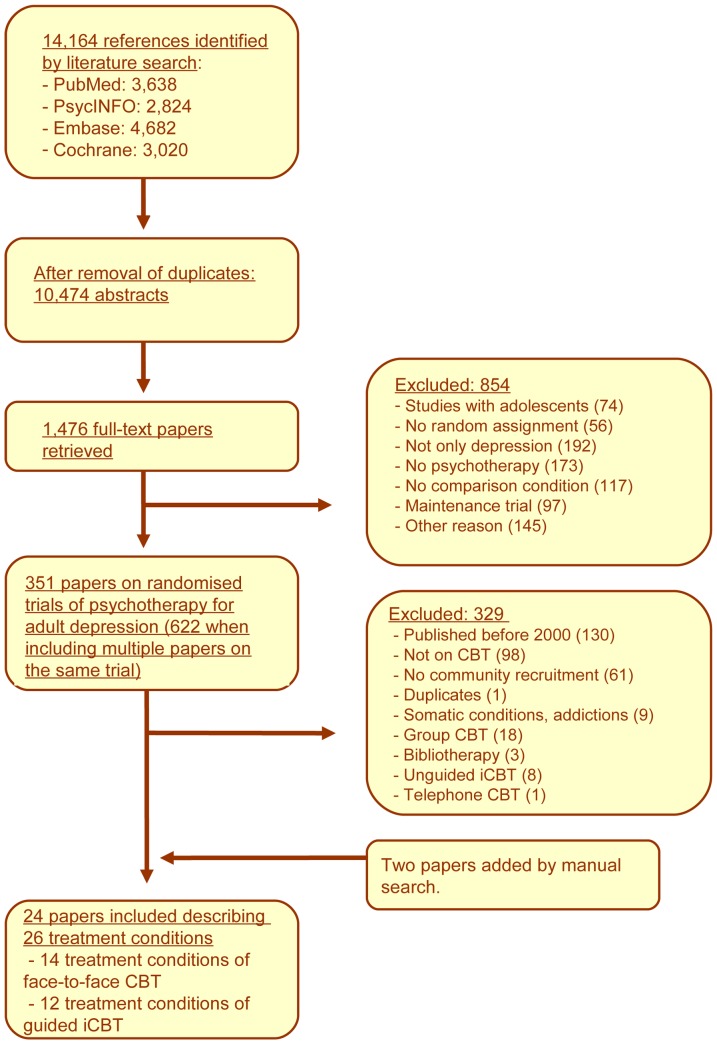
Flow chart of included studies.

Having examined a total of 14,164 abstracts (10,474 after removal of duplicates), we retrieved 1,476 potentially relevant full-text papers for further consideration. We excluded 854 of the retrieved papers (studies with adolescents: 74; no random assignment: 56; included patients who were not depressed: 192; did not meet definition of psychotherapy: 173; no comparison group: 117; maintenance trial: 97; other reason, e.g. insufficient data, protocols, conference abstracts: 145). This resulted in a total of 351 papers on randomised psychotherapy trials of adult depression interventions. Twenty-four studies on individual face-to-face or guided internet-based CBT met inclusion criteria and were included in this study [6]–[8], [32]–[52] (26 treatment groups; 14 individual face-to-face CBT and 12 guided iCBT). See Figure 1 for a flow chart and the specific reasons for exclusion. None of these studies included both a guided iCBT condition and a face-to-face CBT condition. Baseline characteristics of face-to-face CBT groups and guided iCBT groups were comparable in terms of symptom severity. The mean baseline Beck Depression Inventory scores of face-to-face CBT groups ranged from 21.70 to 34.12 (overall mean  =  26.67), while these scores ranged from 19.70 to 28.96 in guided iCBT groups (overall mean  =  25.23).

### Characteristics of the included studies and treatment groups

Six studies on iCBT were conducted in Europe (3 in Sweden, 1 in Switzerland and Germany, 2 in the Netherlands), while 4 were conducted in Australia. Seven studies on face-to-face CBT were conducted in the United States, 2 in Canada, 2 in Germany, 1 in the United Kingdom, 1 in Switzerland and 1 in Romania (Table 1). All but 2 face-to-face studies performed intention-to-treat analyses and all but 2 studies reported the study drop-out rate or sufficient information to infer it (Table 1).

**Table 1:** Included studies.

	Type	N	N completers (100%)	N completers (80%)	N responders to post-treatment	Sessions completed mean (SD)	Maximum amount of sessions	Country	ITT analysis
Bodenmann et al., 2008	FtF CBT	20	20	20	19	20 (0)	20	Swiss	yes
Castonguay et al., 2004	FtF CBT	14	12	NR	12	NR	20	US	no
Constantino et al., 2008	FtF CBT	11	8	8	8	14.38 (2.39)	16	US	yes
David et al., 2008	FtF CBT	56	50	NR	49	17.55 (5.51)^f^	20	Rom	yes
DeRubeis et al., 2005	FtF CBT	60	51	51	51	23.95 (9.72)^f^	28	US	yes
Dimidjian et al., 2006	FtF CBT	45	39	39	39	20.91 (7.97)^f^	24	US	yes
Dunlop et al., 2012	FtF CBT	41	NR	NR	NR	NR	16	US	yes
Hautzinger & Welz, 2008	FtF CBT	31	26	NR	26	NR	15	Ger	yes
McBride et al., 2006	FtF CBT	37	29	NR	28	16.56 (3.22)	20	Can	yes
Serfaty et al., 2009	FtF CBT	70	NR	NR	64	7.09 (4.41)	12	UK	yes
Strauman t al., 2006	FtF CBT	21	17	18	20	18.2 (SD NR)	NR^g^	US	yes
Teismann et al., 2011	FtF CBT	34	33	33	28	22.4 (3.8)	24	Ger	no
Thompson et al., 2001	FtF CBT	31	24	NR	24	NR	20	US	yes
Watson et al., 2003	FtF CBT	NR	33	NR	33	NR	16	Can	yes
Andersson et al., 2005	Guid. iCBT	57	37	NR	36	3.7 (1.9)	5	Swe	yes
Berger et al., 2011	Guid. iCBT	25	14	NR	25	8.52 (2.86)	10	Swiss/Ger	yes
Choi et al., 2012	Guid. iCBT	28	17	17	23	4.93 (1.88)	6	Aus	yes
Johansson et al., 2012^a^	Guid. iCBT	39	NR	NR	36	6.91 (SD NR)	8–10 (m = 9.7)	Swe	yes
Johansson et al., 2012^b^	Guid. iCBT	40	NR	NR	34	5.97 (SD NR)	8	Swe	yes
Perini et al., 2009	Guid. iCBT	27	20	20	18	5.33 (1.21)	6	Aus	yes
Ruwaard et al., 2009	Guid. iCBT	36	33	NR	33	NR	8	NL	yes
Titov et al., 2010^c^	Guid. iCBT	43	33	35	37	5.30 (1.57)	6	Aus	yes
Titov et al., 2010^d^	Guid. iCBT	47	32	37	41	5.21 (1.43)	6	Aus	yes
Titov et al., 2011^e^	Guid. iCBT	18	NR	NR	NR	NR	8	Aus	yes
Vernmark et al., 2010	Guid. iCBT	29	17	NR	27	6 (1.58)	7	Swe	yes

Abbreviations: Guid. iCBT  =  guided internet-delivered cognitive behavioural therapy; FtF CBT  =  individual face-to-face cognitive behavioural therapy;

NR  =  not reported; SD  =  standard deviation; ITT  =  intention to treat.

a Tailored intervention group.

b Standardised intervention group.

c Technician assisted.

d Clinician assisted.

e Transdiagnostic intervention. Adherence was reported, but not specifically for the depressed group.

f Mean and standard deviation estimated.

g Participants completing 12 or more sessions were considered completers, but there was no maximum amount of sessions.

The 26 treatment groups included in our analyses comprised 981 participants who completed pre-treatment measurements and were allocated to CBT. Of these, 504 were allocated to face-to-face CBT and 477 to guided iCBT. One study, which was on face-to-face CBT, did not report the total number of participants who had completed pre-treatment measurements and were allocated to the treatment group. Therefore, this study could not be included in all of the analyses. In seven studies on face-to-face CBT [33], [35], [36], [40]-[42], [44] participants who had not completed the entire treatment but a predefined substantial part of it were considered completers. One study on guided iCBT [48] described an intervention that was individually tailored to the participant and could consist of 8 to 10 sessions. The percentage of completed sessions (mean and standard deviation) was not reported or could not be calculated for 14 groups (9 face-to-face, 5 iCBT). For 4 of these 14 groups (3 face-to-face CBT, 1 iCBT), these variables could be estimated, while it could not be retrieved for the other 10 groups. The percentage of participants who completed 100% of their treatment was not reported for 5 groups (2 face-to-face, 3 iCBT), while for 16 groups the percentage of participants who had completed 80% or more of the treatment was not reported (8 face-to-face, 8 iCBT). When the 80% completion rate was not available, but the 100% completion rate was, the 100% completion data was used. Otherwise, studies with incomplete data were not included in the analyses.

### Adherence

**Figure 2:**
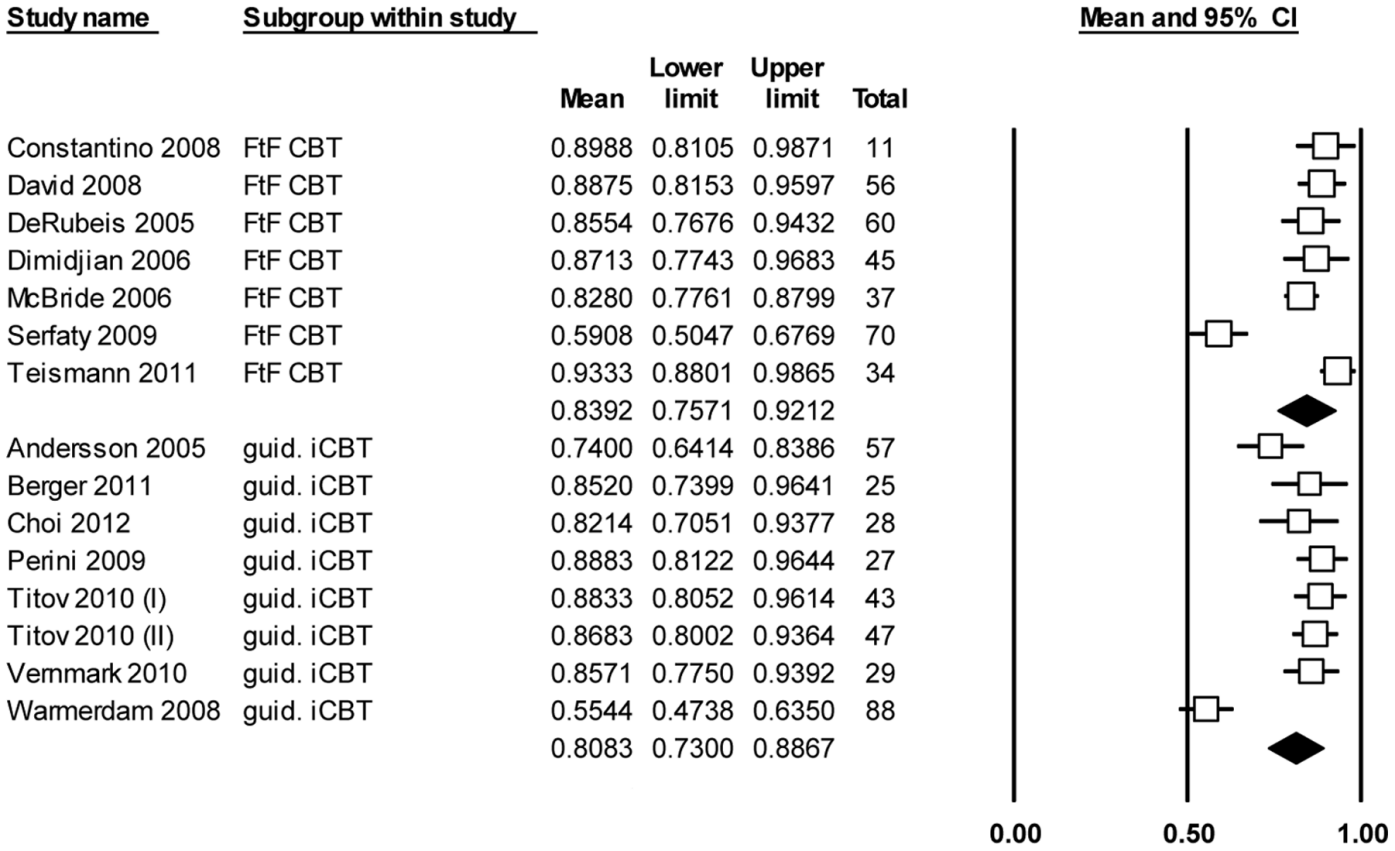
Meta-analysis of percentage of completed sessions, where the completed sessions (mean and sd) are divided by the total number of sessions.

We compared 7 groups of face-to-face CBT and 8 groups of guided iCBT in the analyses of the percentage of completed sessions (Figure 2). One study on face-to-face CBT was excluded from the analysis, because it had a mean completed session rate of 100% with a standard deviation of 0% and could therefore not be analysed. Heterogeneity tests demonstrated significant heterogeneity between studies (*P* < .001, overall *I^2^*  =  86.5%). Face-to-face CBT ranged in length from 12 to 28 sessions. The average percentage of completed face-to-face CBT sessions was 83.9% (CI 75.7% – 92.1%). Guided iCBT interventions ranged in length from 5 to 10 sessions. On average, participants completed 80.8% of their treatments (CI 73.0% – 88.7%). The difference between the percentage of completed sessions for face-to-face CBT and iCBT was not statistically different (*P*  =  .59). See Table 2.

**Table 2:** Meta-analyses of adherence to guided iCBT and individual face-to-face CBT.

	Guided iCBT (95% C.I.)	FtF CBT (95% C.I.)	*P*	*I* ^2^ c
Session completion^a^	80.8% (73.0% – 88.7%)	83.9% (75.7% – 92.1%)	.59	86.5%
Completers (total) b	65.1% (55.3% – 73.8%)	84.7% (78.0% – 89.6%)	< .001	78.4%
Completers (≥ 80%)^b^	67.5% (56.8% – 76.6%)	85.2% (78.1% – 90.4%)	.003	79.3%

a As percentage of the total number of sessions.

b As percentage of all participants who were allocated to the treatment.

c Overall heterogeneity (*Q* value) was significant for all analyses.

Abbreviations: guided iCBT  =  guided internet-delivered cognitive behavioural therapy; FtF CBT  =  individual face-to-face cognitive behavioural therapy; C.I.  =  confidence interval.

**Figure 3:**
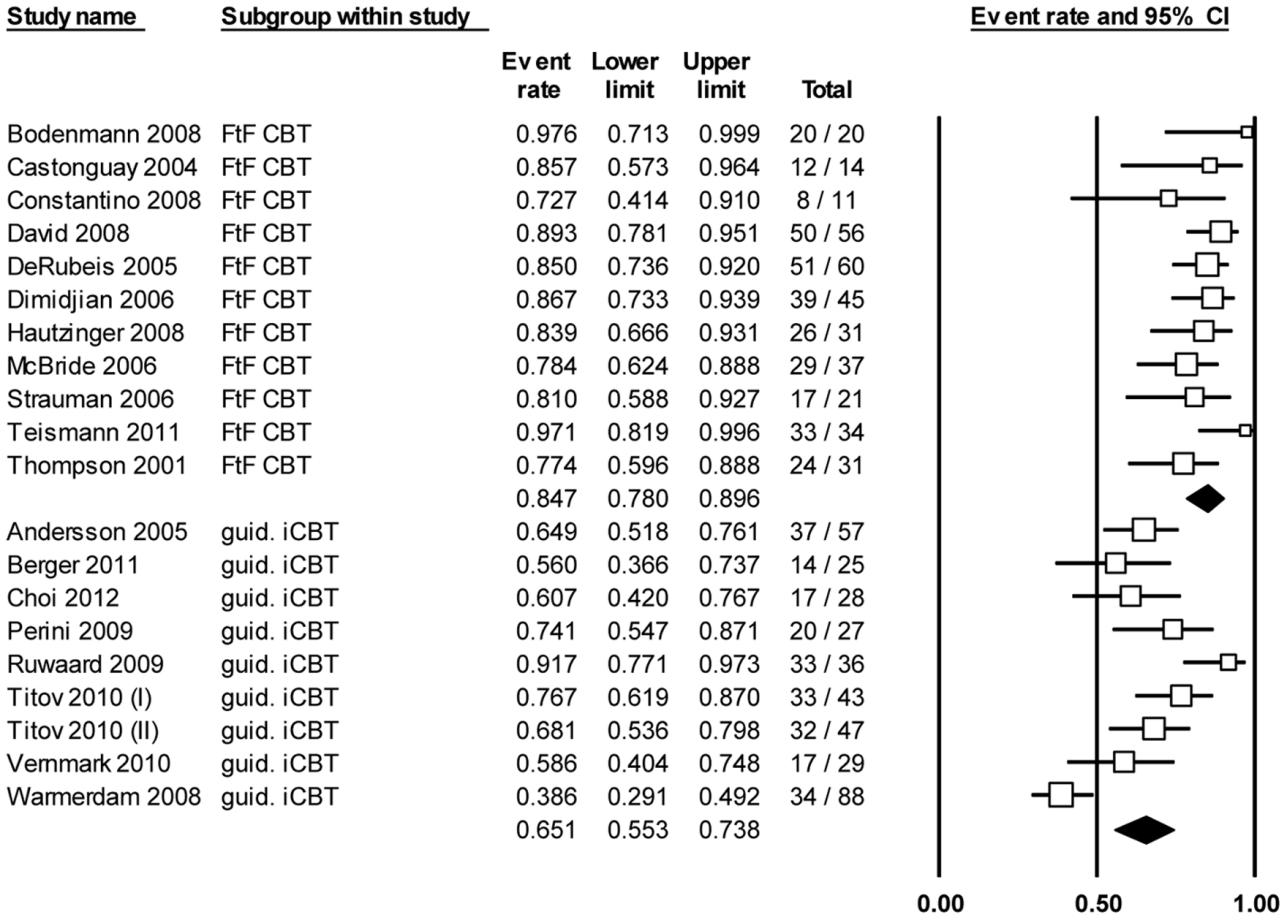
Meta-analysis of percentage of completers of the entire intervention, where N completers is divided by the total N.

In order to compare the percentage of participants who completed the entire intervention (100%), we included 11 face-to-face CBT groups and 9 iCBT groups in our analyses (Figure 3). There was significant heterogeneity between the studies (*P* < .001, *I^2^*  =  78.4%). Of the participants in the face-to-face groups, 84.7% (CI 78.0% – 89.6%) completed the entire intervention. In the guided iCBT groups, this was 65.1% (CI 55.3% – 73.8%). The difference between these rates for face-to-face CBT and guided iCBT was statistically significant (*P* < .001; Table 2).

**Figure 4:**
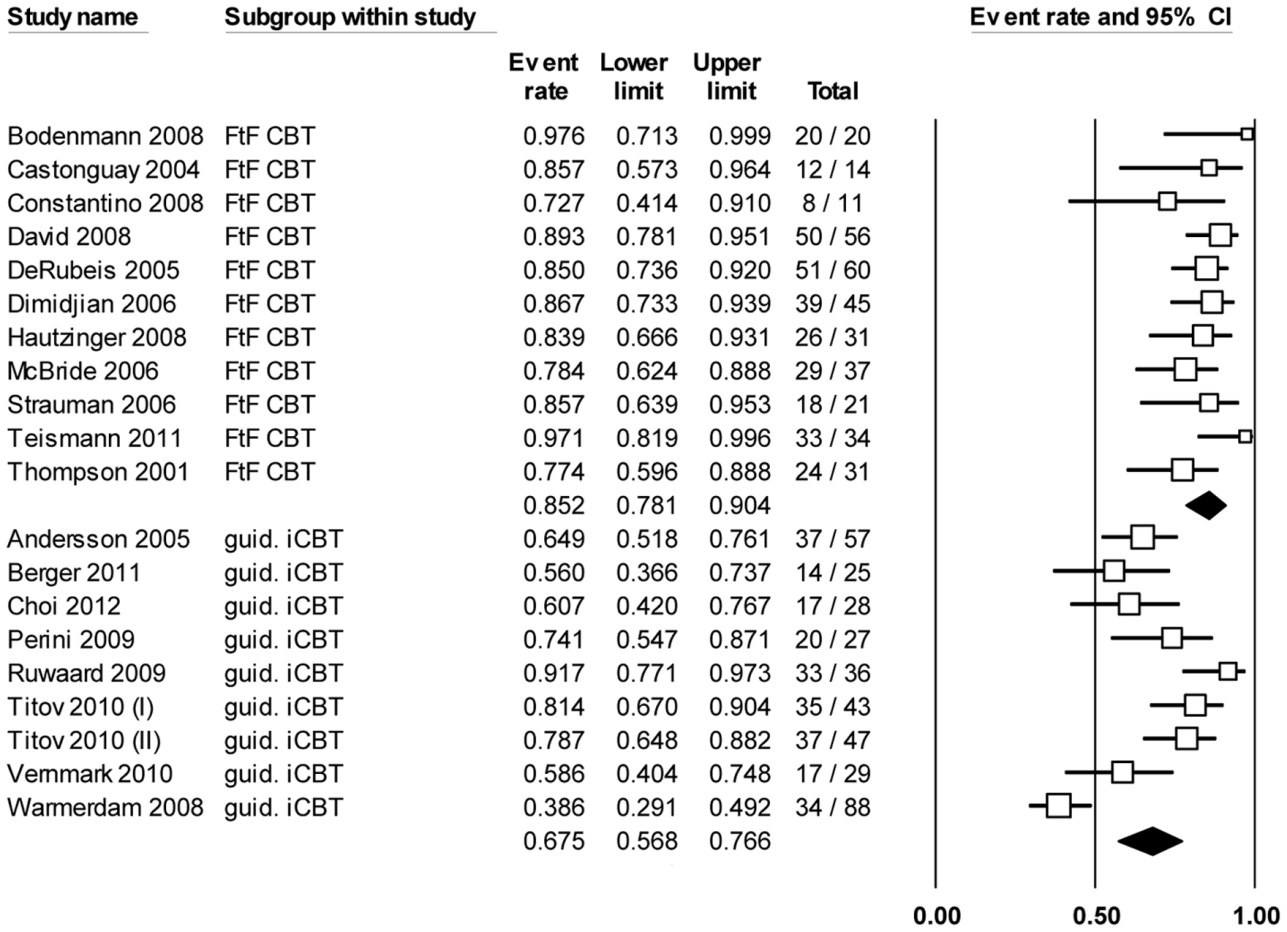
Meta-analysis of the percentage of participants who completed 80% of the sessions, where N completers (80%) is divided by the total N.

Results of the 80% completion analyses, also based on 11 face-to-face CBT groups and 9 iCBT groups, were similar to the 100% completion analyses in terms of overall heterogeneity (*P* < .001, *I^2^*  =  79.3%). The percentage of completers of face-to-face CBT was 85.2% (CI 78.1% – 90.4%), and the percentage of completers of guided iCBT was 67.5% (CI 56.8% – 76.6%). Again, this difference was statistically significant (*P*  =  .003). See Figure 4 and Table 2.

### Adherence of non-completers

For 6 face-to-face CBT groups [34]–[37], [40], [43] and 8 iCBT groups [6]–[8], [46], [47], [49], [51], sufficient information was reported to calculate the adherence of non-completers. Non-completers of face-to-face CBT completed on average 24.5% of their treatment before discontinuing. Non-completers of guided iCBT, on the other hand, completed on average 42.1% of their treatment.

## Discussion

In this meta-analysis, we examined the adherence to guided iCBT, and compared this with the adherence to individual face-to-face CBT. No studies were found that included both a guided iCBT and a face-to-face CBT treatment condition. We analysed 981 participants, who were divided over 26 treatment groups and described by 24 studies. In the guided iCBT groups, participants completed on average 80.8% of their treatment, which did not differ significantly from the face-to-face CBT groups (83.9%). The percentage of treatment completers was higher in face-to-face CBT than in guided iCBT. Of all participants starting face-to-face CBT, 84.7% fully completed their treatment and 85.2% completed at least 80% of their treatment. These numbers were 65.1% and 67.5% for guided iCBT, respectively, which is significantly lower. The discrepancy between these outcomes can be explained by the adherence of non-completers. Non-completers of guided iCBT followed on average 42.1% of their treatment programmes before they dropped out, while non-completers of face-to-face CBT followed 24.5% of their treatment programmes. It should be taken into account that guided iCBT interventions consisted of 5 to 10 sessions, while face-to-face CBT treatments ranged from 12 to 28 sessions.

To date, adherence rates are often expressed as the percentage of a sample that completed an intervention. Looking at this outcome only, our results show a difference between adherence to face-to-face CBT and adherence to guided iCBT. However, the percentage of completed sessions did not differ between face-to-face CBT and iCBT. The percentage of completed sessions may be a more accurate measure of adherence than the percentage of completers, because it gives insight into the adherence of all participants, including the non-completers.

### Adherence to iCBT – comparison with the literature

Richards and Richardson [2] analysed computerised psychological treatments for depression, of which several were iCBT interventions, and found a 65% completion rate for internet interventions with administrative support and 72% for those with therapist-support. Our finding that 65.1% of participants completed the treatment is slightly low in comparison with the study of Richards and Richardson, because we included in our analyses both iCBT groups guided by clinicians and iCBT groups with other guidance. Regarding the percentage of completed sessions, this has not been reported in previous reviews and meta-analyses [2], [14]. Our finding that participants in guided iCBT groups completed on average 80.8% of their treatment therefore cannot be compared with any results in the previous literature.

A different method of analysing drop-out and adherence to internet treatments for psychological disorders was applied in the review by Melville et al. [53], where drop-out rates were calculated at various points in the process of a study, maintaining the number of eligible participants as the denominator. It was found that 21% of eligible participants drop out before commencing treatment and that the same percentage of eligible participants drop-out during the treatment [53]. Depressed mood may be a factor that decreases adherence [53], probably because lack of concentration and motivation are inherent to this condition. Arguably, this method of analysing drop-out and adherence is the most objective and complete, because it gives an overview of attrition in the entire research and intervention process. Adherence and drop-out rates according to this definition are difficult to review, however, because many studies do not supply sufficient information [53].

### Factors associated with adherence to iCBT

Factors that may explain adherence to internet interventions are related to guidance, study design, regularity of updates to the intervention website and persuasive technology (i.e. technology designed to include persuasion and social influence)[17]. With regard to studies on iCBT for depression, the use of persuasive technology and the regularity of updates is usually not reported. Concerning guidance and study design, previous literature does indicate that these factors may affect the adherence to iCBT for depression. Study design has proved to be an important factor, as adherence to treatment in randomised trials is high relative to adherence to open access websites [16]. Guidance is an important factor as well. As previously stated, therapist support leads to better adherence than administrative support in computerised CBT for depression, and both therapist and administrative support lead to better adherence than no support [2]. Previous studies have also shown that guided iCBT is more effective than unguided iCBT [1]. However, when directly comparing therapist support with administrative support in iCBT for depression, in the same trial and using the same intervention, the adherence rates hardly differed and there was no significant difference in clinical outcome or acceptability [6]. Similarly, directly comparing guided iCBT with unguided iCBT for depression, in the same trial and using the same intervention, differences in adherence and clinical effect were small to moderate and not significant [46]. The lack of a difference in clinical effect may be explained by the fact that participants in both arms of the study by Berger et al. had contact with the study team before the treatment started [9]. Both of these studies [6], [46] may have been underpowered. On the other hand, these studies could indicate that differences in adherence and clinical effect are related to the recruited population (e.g. depression symptom severity at baseline) and/or trial design, rather than the amount of guidance. More trials of such direct comparisons would be welcome, in order to gain more insight into the factors that explain adherence to iCBT for depression.

It is often not reported why participants decided to discontinue their treatment. One study on unguided iCBT for depression suggested that drop-outs may have stopped the treatment because they had recovered [54]. It was found that fewer minutes spent on the website and fewer page hits were associated with greater symptom reduction [54]. On the other hand, completion of more sessions of an internet-based intervention was associated with better psychological outcomes in another study [55]. A qualitative study suggested that participants are more likely to complete an internet intervention when they perceive the treatment as beneficial for themselves or for others [22]. Another qualitative study on guided iCBT pointed out that there may be subgroups who only read the instructions provided in the treatment without following them [56], which could indicate that adherence does not always equal compliance with the treatment protocol. More research is needed to understand adherence to internet interventions from the participant's perspective.

### Adherence to face-to-face CBT - comparison with the literature

The adherence rates we found in face-to-face CBT interventions are in line with previous research. Swift and Greenberg [19] found that face-to-face psychotherapy is completed by 80.3% of the participants who start the intervention. Moderators that resulted in adherence rates slightly above average included cognitive behavioural orientation, individual treatment format, and mood disorder as client diagnosis [19]. These findings are in accordance with our finding that 84.7% of participants allocated to individual face-to-face CBT for depression complete their treatment.

### Adherence to face-to-face CBT compared with adherence to iCBT

In terms of clinical effect for depression and anxiety, computerised interventions and face-to-face interventions tend to be equal [57]. Our results indicate that there is also equivalence in the percentage of session completion between guided iCBT and face-to-face CBT. Little attention was given to session completion in previous reviews of studies on internet interventions, except for one [18]. Donkin et al. found correlations between clinical effectiveness and measures of adherence [18]. While the number of logins was correlated with the effectiveness of interventions for health problems, session completion was related to the effectiveness of interventions for depression and anxiety [18]. This finding confirms that if internet-based and face-to-face interventions are equal in terms of effectiveness [57], they are likely to be equal in terms of adherence as well.

Our results suggest that non-completers of face-to-face CBT complete a smaller percentage of their treatment than non-completers of guided iCBT. The absolute number of completed sessions of non-completers is likely to be similar, because face-to-face CBT treatments consist of more sessions. This indicates that participants in face-to-face CBT discontinue their treatment at an early stage of the treatment protocol, but if they have completed several sessions, they are likely to continue until the end. Participants in guided iCBT, on the other hand, drop out more gradually over the course of treatment. The reasons for discontinuing could be quite different between participants who drop out at an earlier stage and participants who drop out at a later stage. If a participant discontinues after only one session, the intervention may not be the kind of treatment he or she was looking for, or it had limited acceptability. If an individual discontinues when he or she is already halfway through, (s)he may have decided the treatment is no longer needed. It is important to note that the guided iCBT interventions in our study are self-help courses. Participants in self-help interventions have more control over their pace and treatment progress than participants in face-to-face therapy, which might explain the difference in adherence behaviour. Additionally, factors such as face-to-face contact and the anonymity of the internet may be of significance.

### Limitations

Our findings should be interpreted with some caution. A first limitation of our study is that we found no study that directly compared guided iCBT with individual face-to-face CBT. The 26 treatment groups we analysed were described by 24 different studies, each with a different design. As has been demonstrated in our results, heterogeneity among studies was large. For example, participants were administered a diagnostic interview in some studies, while other studies included participants based on self-report questionnaires. Studies differed not only by design, but also by their treatment protocol, e.g. one iCBT intervention could be tailored, and seven studies on face-to-face CBT interventions defined treatment completion as a range instead of a fixed number of sessions. The length of the therapy sessions also differed between studies. These factors limit the comparisons in our analyses. Secondly, all face-to-face CBT interventions consisted of more sessions than the guided iCBT interventions. It is not known in what way the length of the programme would affect adherence. Although face-to-face CBT interventions are longer, they have the same content as guided iCBT, and participants have learned the same after completing either of these types of intervention. This is underlined by the equivalence in effectiveness [57]. Thirdly, not all studies reported all adherence rates, so we could not compare all studies in our selection. Some values were estimated to increase the number of studies we could analyse. Although our estimates were intended to be conservative, we may have overestimated the adherence rates of a few studies on face-to-face CBT, as mentioned in the methods section of this paper. Participants in these studies who completed a substantial part of the intervention (e.g. at least twelve of twenty sessions) were defined as completers by the respective studies' authors, while exact numbers of sessions completed were not provided. We counted these participants as full intervention completers for our analyses when other information was lacking. Fourthly, the inclusion criteria of this meta-analysis were designed to include the majority of studies on iCBT and studies on face-to-face CBT that were similar in design. The results of our analyses concerning face-to-face CBT can therefore not be generalised to face-to-face CBT in general. For example, the adherence to face-to-face CBT could be higher in a sample of inpatients. Finally, our results showed a small difference in the mean percentage of completed sessions between face-to-face CBT and guided iCBT. This difference did not reach significance, perhaps because we could not include a sufficiently large number of studies. A future meta-analysis that would include more studies might show whether our results remain confirmed or whether our meta-analysis was underpowered. Still, we can conclude that in terms of completed sessions, there is no difference or only a marginal difference between guided iCBT and face-to-face CBT, at least in community recruited samples.

### Implications and future research

Adherence is an important measure of acceptability, appropriateness, and effect of a psychological treatment. Studies on iCBT and face-to-face CBT should include more detailed information on adherence, preferably both the number of completers and the average number of sessions completed. It is recommended to include clear instructions for reporting adherence rates in the CONSORT statement [58]. Additionally, more research is needed on factors that could explain adherence and the participants' reasons for dropping out. A meta-analysis of individual patient data would enable detailed predictor and moderator analyses. Reasons for dropping out can also be assessed by qualitative studies [22]. Our results would become more meaningful if such data were available.

Although we found no trial that directly compared guided iCBT with individual face-to-face CBT, our results suggest that the adherence to guided iCBT could be adequate compared with the adherence to face-to-face CBT. The adherence to guided iCBT for depression also appears to be adequate relative to other internet interventions. Guided iCBT appears to be an acceptable treatment for depression and efforts should be made to increase its implementation.

## Supporting Information

Checklist S1 PRISMA Checklist.
(DOC)

## References

[1] Andersson G, Cuijpers P (2010) Internet-based and other computerized psychological treatments for adult depression: a meta-analysis. Cogn Behav Ther 38: 196–205. 10.1080/16506070903318960 20183695

[2] Richards D, Richardson T (2012) Computer-based psychological treatments for depression: a systematic review and meta-analysis. Clin Psychol Rev 32: 329–342. 22466510 10.1016/j.cpr.2012.02.004

[3] Christensen H, Griffiths KM, Mackinnon AJ, Brittliffe K (2006) Online randomized controlled trial of brief and full cognitive behaviour therapy for depression. Psychol Med 36: 1737–1746. 16938144 10.1017/S0033291706008695

[4] Clarke G, Eubanks D, Reid E, Kelleher C, O'Connor E, et al (2005) Overcoming Depression on the Internet (ODIN) (2): a randomized trial of a self-help depression skills program with reminders. J Med Internet Res 7: e16. 15998607 10.2196/jmir.7.2.e16 PMC1550641

[5] Meyer B, Berger T, Caspar F, Beevers CG, Andersson G, et al (2009) Effectiveness of a novel integrative online treatment for depression (Deprexis): randomized controlled trial. J Med Internet Res 11: e15. 19632969 10.2196/jmir.1151 PMC2762808

[6] Titov N, Andrews G, Davies M, McIntyre K, Robinson E, et al (2010) Internet treatment for depression: a randomized controlled trial comparing clinician vs. technician assistance. PLoS One 5: e10939. 20544030 10.1371/journal.pone.0010939 PMC2882336

[7] Andersson G, Bergstrom J, Hollandare F, Carlbring P, Kaldo V, et al (2005) Internet-based self-help for depression: randomised controlled trial. Br J Psychiatry 187: 456–461. 16260822 10.1192/bjp.187.5.456

[8] Warmerdam L, van Straten A, Twisk J, Riper H, Cuijpers P (2008) Internet-based treatment for adults with depressive symptoms: randomized controlled trial. J Med Internet Res 10: e44. 19033149 10.2196/jmir.1094 PMC2629364

[9] Johansson R, Andersson G (2012) Internet-based psychological treatments for depression. Expert Rev Neurother 12: 861–869. 22853793 10.1586/ern.12.63

[10] Spek V, Cuijpers P, Nyklicek I, Riper H, Keyzer J, et al (2007) Internet-based cognitive behaviour therapy for symptoms of depression and anxiety: a meta-analysis. Psychol Med 37: 319–328. 17112400 10.1017/S0033291706008944

[11] van Straten A, Cuijpers P, Smits N (2008) Effectiveness of a web-based self-help intervention for symptoms of depression, anxiety, and stress: randomized controlled trial. J Med Internet Res 10: e7. 18364344 10.2196/jmir.954 PMC2483843

[12] Johansson R, Ekbladh S, Hebert A, Lindstrom M, Moller S, et al (2012) Psychodynamic guided self-help for adult depression through the internet: a randomised controlled trial. PLoS One 7: e38021. 22741027 10.1371/journal.pone.0038021 PMC3362510

[13] Gun SY, Titov N, Andrews G (2011) Acceptability of Internet treatment of anxiety and depression. Australas Psychiatry 19: 259–264. 21682626 10.3109/10398562.2011.562295

[14] Kaltenthaler E, Sutcliffe P, Parry G, Beverley C, Rees A, et al (2008) The acceptability to patients of computerized cognitive behaviour therapy for depression: a systematic review. Psychol Med 38: 1521–1530. 18205964 10.1017/S0033291707002607

[15] Gerhards SAH, de Graaf LE, Jacobs LE, Severens JL, Huibers MJH, et al (2010) Economic evaluation of online computerised cognitive-behavioural therapy without support for depression in primary care: randomised trial. Br J Psychiatry 196: 310–318. 20357309 10.1192/bjp.bp.109.065748

[16] Christensen H, Griffiths KM, Farrer L (2009) Adherence in internet interventions for anxiety and depression. J Med Internet Res 11: e13. 19403466 10.2196/jmir.1194 PMC2762797

[17] Kelders SM, Kok RN, Ossebaard HC, Van Gemert-Pijnen JEWC (2012) Persuasive system design does matter: a systematic review of adherence to web-based interventions. J Med Internet Res 14: e152. 23151820 10.2196/jmir.2104 PMC3510730

[18] Donkin L, Christensen H, Naismith SL, Neal B, Hickie IB, et al (2011) A systematic review of the impact of adherence on the effectiveness of e-therapies. J Med Internet Res 13: e52. 21821503 10.2196/jmir.1772 PMC3222162

[19] Swift JK, Greenberg RP (2012) Premature discontinuation in adult psychotherapy: a meta-analysis. J Consult Clin Psychol 80: 547–559. 22506792 10.1037/a0028226

[20] Hatchett GT, Park HL (2003) Comparison of four operational definitions of premature termination. Psychotherapy 40: 226–231.

[21] Eysenbach G (2005) The law of attrition. J Med Internet Res 7: e11. 15829473 10.2196/jmir.7.1.e11 PMC1550631

[22] Donkin L, Glozier N (2012) Motivators and motivations to persist with online psychological interventions: a qualitative study of treatment completers. J Med Internet Res 14: e91. 22743581 10.2196/jmir.2100 PMC3414905

[23] Tierney JF, Stewart LA (2005) Investigating patient exclusion bias in meta-analysis. Int J Epidemiol 34: 79–87. 15561753 10.1093/ije/dyh300

[24] Wierzbicki M, Pekarik G (1993) A meta-analysis of psychotherapy drop-out. Professional Psychology: Research and Practice 24: 190–195.

[25] Mitchell AJ, Selmes T (2007) A comparative survey of missed initial and follow-up appointments to psychiatric specialties in the United kingdom. Psychiatr Serv 58: 868–871. 17535950 10.1176/ps.2007.58.6.868

[26] Waller R, Gilbody S (2009) Barriers to the uptake of computerized cognitive behavioural therapy: a systematic review of the quantitative and qualitative evidence. Psychol Med 39: 705–712. 18812006 10.1017/S0033291708004224

[27] Cuijpers P, van Straten A, Warmerdam L, Andersson G (2008) Psychological treatment of depression: a meta-analytic database of randomized studies. BMC Psychiatry 8: 36. 18485191 10.1186/1471-244X-8-36 PMC2408566

[28] Beck AT, Rush AJ, Shaw BF, Emery G (1979) Cognitive therapy of depression. New York: Guilford Press.

[29] Lewinsohn PM, Antonucci DO, Breckenridge JS, Teri L (1984) The "Coping With Depression" course. Eugene, OR: Castalia.

[30] Higgins JPT, Green S (2008) Cochrane handbook for systematic reviews of interventions. (Version 5.0.1). The Cochrane Collaboration.

[31] Higgins JPT, Thompson SG, Deeks JJ, Altman DG (2003) Measuring inconsistency in meta-analyses. BMJ 327: 557–560. 12958120 10.1136/bmj.327.7414.557 PMC192859

[32] Bodenmann G, Plancherel B, Beach SRH, Widmer K, Gabriel B, et al (2008) Effects of coping-oriented couples therapy on depression: a randomized clinical trial. J Consult Clin Psychol 76: 944–954. 19045963 10.1037/a0013467

[33] Castonguay LG, Schutt AJ, Aikins DE, Constantino MJ, Laurenceau JP, et al (2004) Integrative cognitive therapy for depression: A preliminary investigation. Journal of Psychotherapy Integration 14: 4–20.

[34] Constantino MJ, Marnell ME, Haile AJ, Kanther-Sista SN, Wolman K, et al (2008) Integrative cognitive therapy for depression: A randomized pilot comparison. Psychotherapy (Chic) 45: 122–134. 22122413 10.1037/0033-3204.45.2.122

[35] David D, Szentagotai A, Lupu V, Cosman D (2008) Rational emotive behavior therapy, cognitive therapy, and medication in the treatment of major depressive disorder: a randomized clinical trial, posttreatment outcomes, and six-month follow-up. J Clin Psychol 64: 728–746. 18473339 10.1002/jclp.20487

[36] DeRubeis RJ, Hollon SD, Amsterdam JD, Shelton RC, Young PR, et al (2005) Cognitive therapy vs medications in the treatment of moderate to severe depression. Arch Gen Psychiatry 62: 409–416. 15809408 10.1001/archpsyc.62.4.409

[37] Dimidjian S, Hollon SD, Dobson KS, Schmaling KB, Kohlenberg RJ, et al (2006) Randomized trial of behavioral activation, cognitive therapy, and antidepressant medication in the acute treatment of adults with major depression. J Consult Clin Psychol 74: 658–670. 16881773 10.1037/0022-006X.74.4.658

[38] Dunlop BW, Kelley ME, Mletzko TC, Velasquez CM, Craighead WE, et al (2012) Depression beliefs, treatment preference, and outcomes in a randomized trial for major depressive disorder. J Psychiatr Res 46: 375–381. 22118808 10.1016/j.jpsychires.2011.11.003 PMC3288535

[39] Hautzinger M, Welz S (2008) Short- and long-term efficacy of psychological intervention for depression in older adults. Zeitschrift Fur Klinische Psychologie Und Psychotherapie 37: 52–60.

[40] McBride C, Atkinson L, Quilty LC, Bagby RM (2006) Attachment as moderator of treatment outcome in major depression: a randomized control trial of interpersonal psychotherapy versus cognitive behavior therapy. J Consult Clin Psychol 74: 1041–1054. 17154734 10.1037/0022-006X.74.6.1041

[41] Serfaty MA, Haworth D, Blanchard M, Buszewicz M, Murad S, et al (2009) Clinical effectiveness of individual cognitive behavioral therapy for depressed older people in primary care: a randomized controlled trial. Arch Gen Psychiatry 66: 1332–1340. 19996038 10.1001/archgenpsychiatry.2009.165

[42] Strauman TJ, Vieth AZ, Merrill KA, Kolden GG, Woods TE, et al (2006) Self-system therapy as an intervention for self-regulatory dysfunction in depression: a randomized comparison with cognitive therapy. J Consult Clin Psychol 74: 367–376. 16649881 10.1037/0022-006X.74.2.367

[43] Teismann T, Dymel W, Schulte D, Willutzki U (2011) [Resource-focused treatment for unipolar depression: a randomized controlled psychotherapy study]. Psychother Psychosom Med Psychol 61: 295–302. 21337296 10.1055/s-0030-1270453

[44] Thompson LW, Coon DW, Gallagher-Thompson D, Sommer BR, Koin D (2001) Comparison of desipramine and cognitive/behavioral therapy in the treatment of elderly outpatients with mild-to-moderate depression. Am J Geriatr Psychiatry 9: 225–240. 11481130

[45] Watson JC, Gordon LB, Stermac L, Kalogerakos F, Steckley P (2003) Comparing the effectiveness of process-experiential with cognitive-behavioral psychotherapy in the treatment of depression. J Consult Clin Psychol 71: 773–781. 12924682 10.1037/0022-006x.71.4.773

[46] Berger T, Hammerli K, Gubser N, Andersson G, Caspar F (2011) Internet-based treatment of depression: a randomized controlled trial comparing guided with unguided self-help. Cogn Behav Ther 40: 251–266. 22060248 10.1080/16506073.2011.616531

[47] Choi I, Zou J, Titov N, Dear BF, Li S, et al (2012) Culturally attuned Internet treatment for depression amongst Chinese Australians: a randomised controlled trial. J Affect Disord 136: 459–468. 22177742 10.1016/j.jad.2011.11.003

[48] Johansson R, Sjoberg E, Sjogren M, Johnsson E, Carlbring P, et al (2012) Tailored vs. standardized internet-based cognitive behavior therapy for depression and comorbid symptoms: a randomized controlled trial. PLoS One 7: e36905. 22615841 10.1371/journal.pone.0036905 PMC3352859

[49] Perini S, Titov N, Andrews G (2009) Clinician-assisted Internet-based treatment is effective for depression: randomized controlled trial. Aust N Z J Psychiatry 43: 571–578. 19440890 10.1080/00048670902873722

[50] Ruwaard J, Schrieken B, Schrijver M, Broeksteeg J, Dekker J, et al (2009) Standardized web-based cognitive behavioural therapy of mild to moderate depression: a randomized controlled trial with a long-term follow-up. Cogn Behav Ther 38: 206–221. 19221919 10.1080/16506070802408086

[51] Vernmark K, Lenndin J, Bjarehed J, Carlsson M, Karlsson J, et al (2010) Internet administered guided self-help versus individualized e-mail therapy: A randomized trial of two versions of CBT for major depression. Behav Res Ther 48: 368–376. 20152960 10.1016/j.brat.2010.01.005

[52] Titov N, Dear BF, Schwencke G, Andrews G, Johnston L, et al (2011) Transdiagnostic internet treatment for anxiety and depression: a randomised controlled trial. Behaviour research and therapy 49: 441–452. 21679925 10.1016/j.brat.2011.03.007

[53] Melville KM, Casey LM, Kavanagh DJ (2010) Dropout from Internet-based treatment for psychological disorders. Br J Clin Psychol 49: 455–471. 19799804 10.1348/014466509X472138

[54] Clarke G, Kelleher C, Hornbrook M, Debar L, Dickerson J, et al (2010) Randomized effectiveness trial of an Internet, pure self-help, cognitive behavioral intervention for depressive symptoms in young adults. Cogn Behav Ther 38: 222–234. 10.1080/16506070802675353 PMC2829099 19440896

[55] Christensen H, Griffiths K, Groves C, Korten A (2006) Free range users and one hit wonders: community users of an Internet-based cognitive behaviour therapy program. Aust N Z J Psychiatry 40: 59–62. 16403040 10.1080/j.1440-1614.2006.01743.x

[56] Bendelin N, Hesser H, Dahl J, Carlbring P, Nelson KZ, et al (2011) Experiences of guided Internet-based cognitive-behavioural treatment for depression: a qualitative study. BMC Psychiatry 11: 107. 21718523 10.1186/1471-244X-11-107 PMC3142491

[57] Cuijpers P, Donker T, van Straten A, Li J, Andersson G (2010) Is guided self-help as effective as face-to-face psychotherapy for depression and anxiety disorders? A systematic review and meta-analysis of comparative outcome studies. Psychol Med 40: 1943–1957. 20406528 10.1017/S0033291710000772

[58] Schulz KF, Altman DG, Moher D (2010) for the CONSORT Group (2010) CONSORT 2010 Statement: Updated Guidelines for Reporting Parallel Group Randomised Trials. PLoS Med 7(3): e1000251. 20352064 10.1371/journal.pmed.1000251 PMC2844794

